# PTRH2 is Necessary for Purkinje Cell Differentiation and Survival and its Loss Recapitulates Progressive Cerebellar Atrophy and Ataxia Seen in IMNEPD Patients

**DOI:** 10.1007/s12311-022-01488-z

**Published:** 2022-10-11

**Authors:** Sylvie Picker-Minh, Ilaria Luperi, Ethiraj Ravindran, Nadine Kraemer, Sami Zaqout, Gisela Stoltenburg-Didinger, Olaf Ninnemann, Luis R. Hernandez-Miranda, Shyamala Mani, Angela M. Kaindl

**Affiliations:** 1 Department of Pediatric Neurology, Charité—Universitätsmedizin Berlin, Augustenburger Platz 1, 13353 Berlin, Germany; 2 Institute of Cell- and Neurobiology, Charité—Universitätsmedizin Berlin, Charitéplatz 1, 10117 Berlin, Germany; 3 Center for Chronically Sick Children (Sozialpädiatrisches Zentrum, SPZ), Charité—Universitätsmedizin Berlin, Augustenburger Platz 1, 13353 Berlin, Germany; 4 Berlin Institute of Health, Berlin, Germany; 5 Department of Basic Medical Sciences, College of Medicine, QU Health, Qatar University, Doha, Qatar; 6 Biomedical and Pharmaceutical Research Unit, QU Health, Qatar University, Doha, Qatar

**Keywords:** PTRH2, Purkinje cells, Ataxia, Cell survival, mTOR, IMNEPD

## Abstract

Hom ozygous variants in the peptidyl-tRNA hydrolase 2 gene (*PTRH2*) cause infantile-onset multisystem neurologic, endocrine, and pancreatic disease. The objective is to delineate the mechanisms underlying the core cerebellar phenotype in this disease. For this, we generated constitutive (*Ptrh2LoxPxhCMVCre, Ptrh2*^*−/−*^* mice*) and Purkinje cell (PC) specific (*Ptrh2LoxPxPcp2Cre, Ptrh2*^*ΔPC*^*mice*) *Ptrh2* mutant mouse models and investigated the effect of the loss of *Ptrh2* on cerebellar development. We show that *Ptrh2*^*−/−*^ knockout mice had severe postnatal runting and lethality by postnatal day 14. *Ptrh2*^*ΔPC*^ PC specific knockout mice survived until adult age; however, they showed progressive cerebellar atrophy and functional cerebellar deficits with abnormal gait and ataxia. PCs of *Ptrh2*^*ΔPC*^ mice had reduced cell size and density, stunted dendrites, and lower levels of ribosomal protein S6, a readout of the mammalian target of rapamycin pathway. By adulthood, there was a marked loss of PCs. Thus, we identify a cell autonomous requirement for PTRH2 in PC maturation and survival. Loss of PTRH2 in PCs leads to downregulation of the mTOR pathway and PC atrophy. This suggests a molecular mechanism underlying the ataxia and cerebellar atrophy seen in patients with *PTRH2* mutations leading to infantile-onset multisystem neurologic, endocrine, and pancreatic disease.

**Supplementary Information:**

The online version contains supplementary material available at 10.1007/s12311-022-01488-z.

## Introduction

Infantile-onset multisystem neurologic, endocrine, and pancreatic disease (IMNEPD) (IMNEPD; MIM#616,263) was reported recently as a novel disease entity that affects multiple organs including a nervous system phenotype. The disease is caused by homozygous variants in the peptidyl-tRNA hydrolase 2 (*PTRH2*) gene, and since the original report [1] several cases have been described with phenotypes varying from mild to more severe forms [2, 3]. All IMNEPD cases have intellectual and motor disability, and most patients show ataxia accompanied by cerebellar atrophy. The pathomechanism underlying the cerebellar atrophy is unknown, and to date there is no treatment.

PTRH2 is widely expressed in various cell types, and both IMNEPD patients and *Ptrh2* mutant mice show severe growth retardation. In the developing cerebellum, PTRH2 is expressed in postmitotic cells including the Purkinje cells (PCs) [1]. In the adult cerebellum, PTRH2 expression is specific to the PC bodies and the dendrites in the molecular layer. Our earlier analysis in the *Ptrh2-Meox-Cre* mice showed a decrease in cerebellar area suggesting that *Ptrh2* plays a role in cerebellar development [1]. The *Ptrh2-Meox-Cre* mice were generated by crossing *Ptrh2*^*flox/flox*^ mice with mice that expressed Cre recombinase under the control of the endogenous Meox2 promoter [4]. These mice also showed runting and died in the second postnatal week. We previously linked the growth retardation phenotype to a decreased activation of the mTOR pathway and reported decreased levels of phosphorylated ribosomal protein S6 (pS6), an indicator of mTOR activation levels in patient cells and *Ptrh2-Meox-Cre* mice [1]. mTOR is a major regulator of mass accumulation and important for regulating cell growth [5, 6]. Moreover, the mTOR pathway has been shown to be critically important for the survival and differentiation of PCs [7–9], which points to a putative mechanism linking *PTRH2* dysfunction to the cerebellar IMNEPD phenotype.

PC axons constitute the sole output of the cerebellar cortex, and an abnormal PC phenotype often underlies ataxia, a core symptom of IMNEPD [10]. PCs are born in the ventricular zone (VZ) between E10 and E12.5, migrate out and around E18, and begin to express sonic hedgehog (SHH), a major mitogen for the granule neuron precursor (GNP) proliferation [11, 12]. The first postnatal week is the peak for GNP proliferation during which time the PCs disperse into a monolayer. PCs develop an elaborate dendritic structure and establish synaptic connections in the second and third postnatal week [13, 14]. Since PC development is ongoing during the time when *Ptrh2* null mice are dying, the question of whether PTRH2 has a cell autonomous role to play in PC maturation and survival could not be addressed and remains an open question.

In this study, we investigate the role of *Ptrh2* in PC differentiation and survival and its effect on cerebellum development and homeostasis.

## Materials and Methods

### Generation of Constitutive *Ptrh2* and Conditional *Ptrh2* Mutant Mice

Constitutive *Ptrh2*-mutant mice were generated by breeding *Ptrh2* LoxP^+/+^ mice (*C57BL*/6-*Ptrh2*^*tm1Eruo*^/J, Jackson Lab #010,711) with *hCMV-cre* to obtain heterozygous *Ptrh2 LoxP*^+*/−*^* hCMV-cre* ^+*/−*^ mice, which were further intercrossed to obtain the *Ptrh2* mutant mice *Ptrh2 LoxP*^+*/*+^*hCMV-cre* ^+*/−*^. *C57BL/6-hCMV-cre* mice were obtained from the animal facility of the Charité—Universitätsmedizin Berlin, Germany. Genotyping was performed using the primers G2F 5′-TGG GTC TTT GAATCA ACT AG-3′, G1R 5′-ACA TGC CAC AAG CAA CTC CA-3′, and 30d 5′-TTT GAG ACC CTA TCA CTC CAC ACG-3′ with an expected 250 bp band in wild-type, a 200 bp band in homozygous *Ptrh2* mutant mice, and both bands in heterozygous mice, as previously described [1, 15].

*Conditional Ptrh2*-mutant mice were generated by breeding *Ptrh2* LoxP^+/+^ mice (*C57BL*/6-*Ptrh2*^*tm1Eruo*^/J, Jackson Lab #010,711) with the L7/*PcP2*:Cre (received from Dr. Kaether, [16]) to obtain heterozygous *Ptrh2 LoxP*^+*/−*^ PcP2-c*re*^+*/−*^ mice, which were further intercrossed to obtain the *Ptrh2* mouse line *Ptrh2 LoxP*^+*/*+^*PcP2-cre* ^+*/−*^. Genotyping was performed using the two primer combinations: (i) G2F 5′-TGG GTC TTT GAA TCA ACT AG-3′and G1R 5′-ACA TGC CAC AAG CAA CTC CA-3′ to detect the *Ptrh2* LoxP site with an expected 250 bp band in wild type, a 300 bp band in homozygous *Ptrh2*-floxed mice, and both bands in heterozygous mice; (ii) Cre1 5′-CGG TCG ATG CAA CGA GTG ATG-3′ and Cre2 5′-CCA GAG ACG GAA ATC CAT CGC-3′ to detect the *Pcp2* Cre with an expected size of 643 bp.

Viability and breeding of various genotypes (wild type [WT], heterozygous, homozygous) of *Ptrh2* mice were monitored continuously. The breeding was performed during the day, the day of insemination was considered as embryonic day (E) 0, and the day of birth was designated as postnatal day (P) 0.

For further analysis *Ptrh2* knockout mice and control littermates were sacrificed at P0, P4 or P5, and their organs snap-frozen for RNA and protein extraction and/or immersed in 4% paraformaldehyde for histological analysis as reported previously [17]. Brain was embedded in paraffin and analyzed following hematoxylin and eosin staining of 10-μm sections. mRNA and protein levels were analyzed through quantitative real-time PCR and western blotting, respectively.

### Quantitative Real-Time PCR (qPCR)

RNA extraction and cDNA synthesis were performed with established methods reported previously [18]. To specifically amplify and detect *Shh*, *Gli1 (GLI family zinc protein 1)*, *Gli2*, and *Hprt* (Hypoxanthin-Guanin-Phosphoribosyltransferase, reference gene) cDNA, we designed sets of primers using the Primer3 online software (www.primer3.ut.ee). qPCR experiments were run in triplicates using Maxima SYBR Green/ROX qPCR Master Mix for *Gli1* and *Gli2* (Thermo Scientific, Braunschweig, Germany) and with a specific probe for *Shh* according to the manufacturer’s protocol. Quantification was performed as described previously [18], and statistical calculations were performed using the GraphPad Prism 8 Software (GraphPad Software Inc., La Jolla, CA, USA). Primer pairs used are as follows: *Gli1* F: GCTGCACTGAAGGATCTCTCT, *Gli1* R: TTACGGTTTGCAGGTCGAGG; *Gli2* F: CAGCCTTCACTTTTCCCCAC, *Gli2* R: GAGGTGAGAGTCATGGGCTG; *Shh* F: GCTGATGACTCAGAGGTGCAAA, *Shh* R: CCTCAGTCACTCGAAGCTTCACT, *HPRT* F: ATCATTATGCCGAGGATTTGGAA, *HPRT* R: TTGAGCACACAGAGGGCCA.

### Western Blot

Protein extraction and western blots (run in triplicates) were performed with established methods reported previously [19]. Antibodies are listed in Table 1.

**Table 1:** Antibody list and dilutions

AB list WB				
	**Host**	**Company**	**Cat. No**	**dilution**
pS6	rb	Cell Signaling	48,565	1:1000
Shh	ms	Abcam	135,240	135,240
Vinculin	ms	Sigma	031M4818	1:10,000
Actin	ms	Millipore	1:1000	1:1000
AB list IHC				
	**Host**	**Company**	**Cat. No**	**dilution**
Calbindin	ms	Swant	CB300	1:2000
cCasp3	rb	Cell Signaling	9661S	1:200
FoxP4	rb	Millipore	ABE74	1:500
GFAP	chicken	Abcam	ab4674	1:6000
Gli1	rb	Thermo Scientific	PA5-32,206	1:200
Ki67	rb	Zytomed	RBK027	1:100
Mbh2	rb	Novus Biologicals	NBP1-86,513	1:400
pS6	rb	Cell Signaling	48,565	1:200
SHH	ms	Abcam	135,240	1:400
Tlx3	gp	Gift from Dr. Hernandez-Miranda, Charite		1:5000
Secondary Abs				
	**Host**	**Company**	**Cat. No**	**dilution**
Donkey anti-Mouse IgG Alexa Fluor 488	donkey	Thermo Scientific	A-21202	1:400
Donkey anti-Mouse IgM Cy3	donkey	Jackson	715–165-020	1:400
Donkey anti-Rabbit IgG Alexa Fluor 488	donkey	Thermo Scientific	A-21206	1:400
Donkey anti-Rabbit IgG Cy3	donkey	Jackson	711–165-152	1:400
Donkey anti-Rat IgG Alexa Fluor 488	donkey	Thermo Scientific	A-21208	1:400
Goat anti-Chicken IgG Alexa Fluor 488	goat	Thermo Scientific	A-11039	1:400
DAPI		Sigma	28,718–90-3	1:1000

### Mixed Cerebellar Culture and Immunocytology

Mixed cerebellar neuron cultures were prepared as previously published [20, 21]. Coverslips with cerebellar neuron cultures were fixed in 4% paraformaldehyde.

### Immunohistology

Paraffin sections were deparaffinized prior to rinsing. Coverslips and paraffin sections were further incubated in staining buffer (0.2% gelatin, 0.25% Triton X-100, 10% donkey normal serum) for 30 min for permeabilization and blocking, followed by overnight incubation with primary antibodies and a 2-h incubation with the corresponding secondary antibodies (antibodies listed in Table1). Nuclei were labeled with 4′,6-diamidino-2-phenylindole (DAPI, 1:1000, Sigma-Aldrich). Fluorescence staining was analyzed and imaged using a fluorescent Olympus BX51 microscope with the software Magnafire 2.1B (2001) (Olympus, Hamburg, Germany), and images were processed using Adobe Photoshop and ImageJ.

### Ki67 Analysis

Images were taken across all lobes including the gyri and sulci areas under 40 × magnification using the Olympus BX51 microscope. In the ImageJ image processing program (RRID:SCR_003070), using the toolbox, the external granule layer (EGL) extent was outlined using DAPI, and the area was measured. The number of Ki67 positive cells within that area were counted, and the number of cells counted was divided by the EGL area to obtain number of cells per unit area.

### Golgi Staining 

Golgi staining was performed as previously described by our group [22].

### Ataxia Test

A simple composite phenotype scoring system including scoring of a ledge test, hindlimb clasping, gait, and kyphosis was performed on cerebellar knockout mice at 6 months age and 18 months age as previously published [23]. Scores for each test parameter were added as previously published [23].

### Footprint Analysis

A footprint analysis was performed on cerebellar knockout mice at 6 months age in collaboration with the Animal Outcome Core facility of the Charité—Universitätsmedizin Berlin. A footprint apparatus was used, which consisted of a short runway with white paper with a goal-box at the end of the runway. On days prior to the experiment, mice were trained to walk toward the goal-box. For the footprint analysis, forepaws were painted in red and hindpaws in green and set free to walk on the runway. The footprint patterns were analyzed for stride length, forebase/hindbase width, and forepaw/hindpaw overlap.

## Statistics

All pictures shown are representative. Histograms are plotted as mean + / − SD except where indicated. Student’s *t* test was used to test for statistical significance. False discovery rate in the case of multiple *t* test was corrected by the Benjamini and Yakutiel method (GraphPad Prism). The number of animals per group used for statistical analysis and the significance values are given in the figure legends.

## Results

We previously demonstrated cerebellar atrophy in human IMNEPD patients and *Ptrh2* mutant mice as well as a strong expression of PTRH2 in murine cerebellar postmitotic PCs [1]. To investigate the function of PTRH2 in both early and late phases of PC development, we generated two mouse lines, a constitutive *Ptrh2* knockout mouse (*Ptrh2LoxPxhCMVCre*, subsequently abbreviated as *Ptrh2*^*−/−*^) and a PC specific conditional *Ptrh2* KO mouse (*Ptrh2LoxPxPcp2Cre*, subsequently abbreviated as *Ptrh2*^ΔPC^ mouse). We used the *Ptrh2*^*−/−*^ mouse model to analyze the consequences for cerebellar development at early postnatal stages and the *Ptrh2*^ΔPC^ mouse to investigate the cell autonomous role of PTRH2 in PCs and consequences for cerebellar structure and function in adult mice.

### *Ptrh2*.^*−/−*^Mice Are Normal at P0 but Have Reduced Cerebellar Volume and Folia Formation at P5

**Fig. 1:**
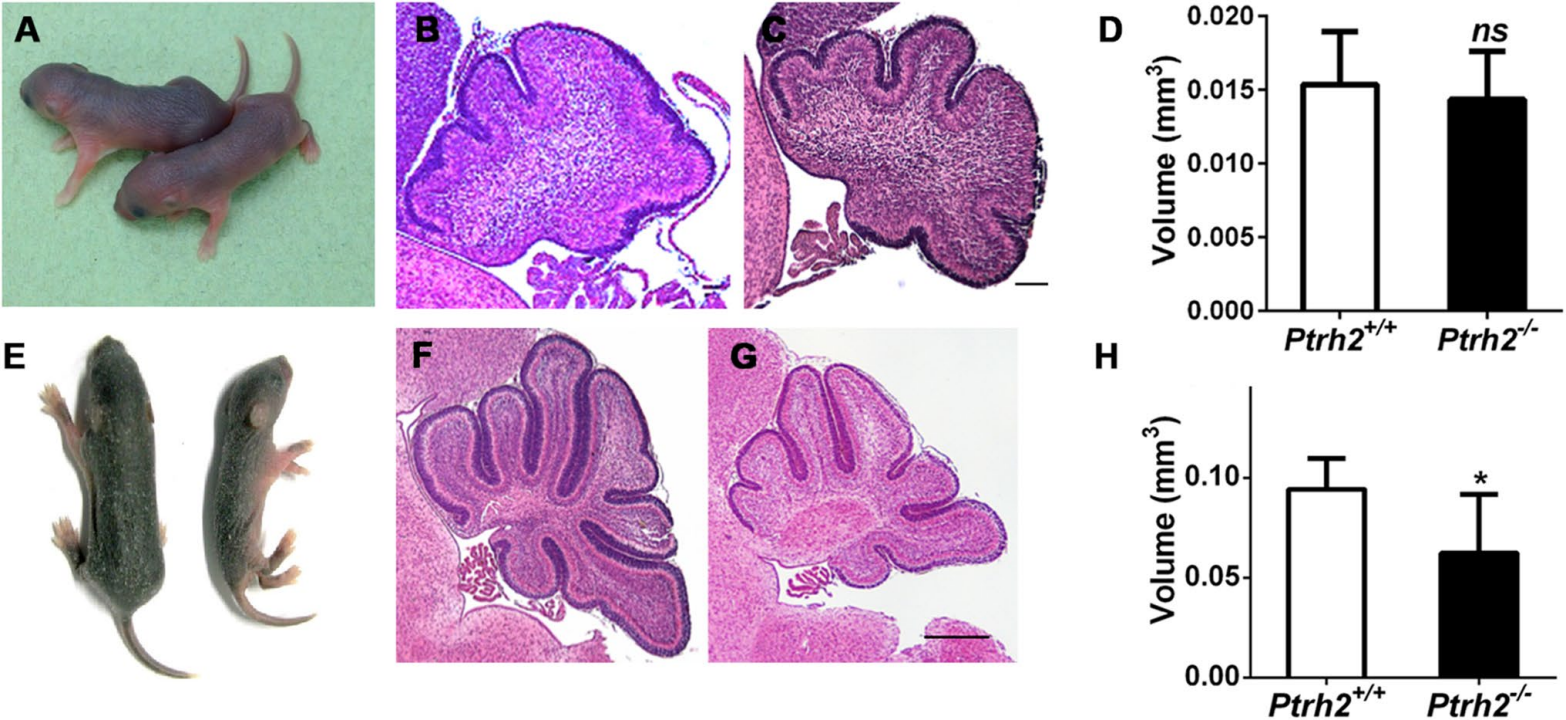
*Ptrh2*^*−/−*^ mice are normal at birth but at P5 show runting syndrome and impaired cerebellar development (**A**–**D** = P0) (**E**–**H** = P5) (**A**) P0 pup *Ptrh2* ^+/+^ (left) and *Ptrh2*^*−/−*^ (right). P0 mid-sagittal cerebellum section from (**B**) *Ptrh2* ^+/+^ and (**C**) *Ptrh2*^*−/−*^ mice (HE, scalebar 200 µm). (**D**) Quantitation of cerebellar volume at P0. (**E**) P5 pups, *Ptrh2* ^+/+^ (left) and *Ptrh2*^*−/−*^ (right). P5 mid-sagittal cerebellum section from (**F**) *Ptrh2* ^+/+^ and (**G**) *Ptrh2*.^*−/−*^ mice (HE, scalebar 200 µm). (**H**) Quantitation of cerebellar volume at P5. Quantitation based on *n* = 6 animals/group. *ns* not significant, * *p* < 0.05

Directly after birth at P0, *Ptrh2*^*−/−*^ mice were phenotypically indistinguishable from their wild-type littermates (*Ptrh2*^+^*/*^+^ mice) (Fig. 1A). At P0, cerebella of *Ptrh2*^*−/−*^ and *Ptrh2*^+*/*+^ mice were indistinguishable and showed the beginning of cardinal cerebellar foliae formation in mid-sagittal sections (Fig. 1B, C). There was no difference in cerebellar volume at that age (Fig. 1D). *Ptrh2*^*−/−*^ mice developed a runting syndrome postnatally (Fig. 1E) and died within the first two postnatal weeks, as reported earlier [1, 15]. While formation of cerebella foliae had progressed significantly in *Ptrh2*^+*/*+^ mice at P5 (Fig. 1F) they were considerably shallower in *Ptrh2*^*−/−*^ mice (Fig. 1G). At P5 *Ptrh2*^*−/−*^ mice also showed significantly reduced cerebellar volume (Fig. 1H). We next investigated the changes at the cellular level that could account for these gross structural changes.

### *Ptrh2*.^*−/−*^ Mice Show Reduced Proliferation in the EGL and Reduced Neurons in the IGL at P5

**Fig. 2:**
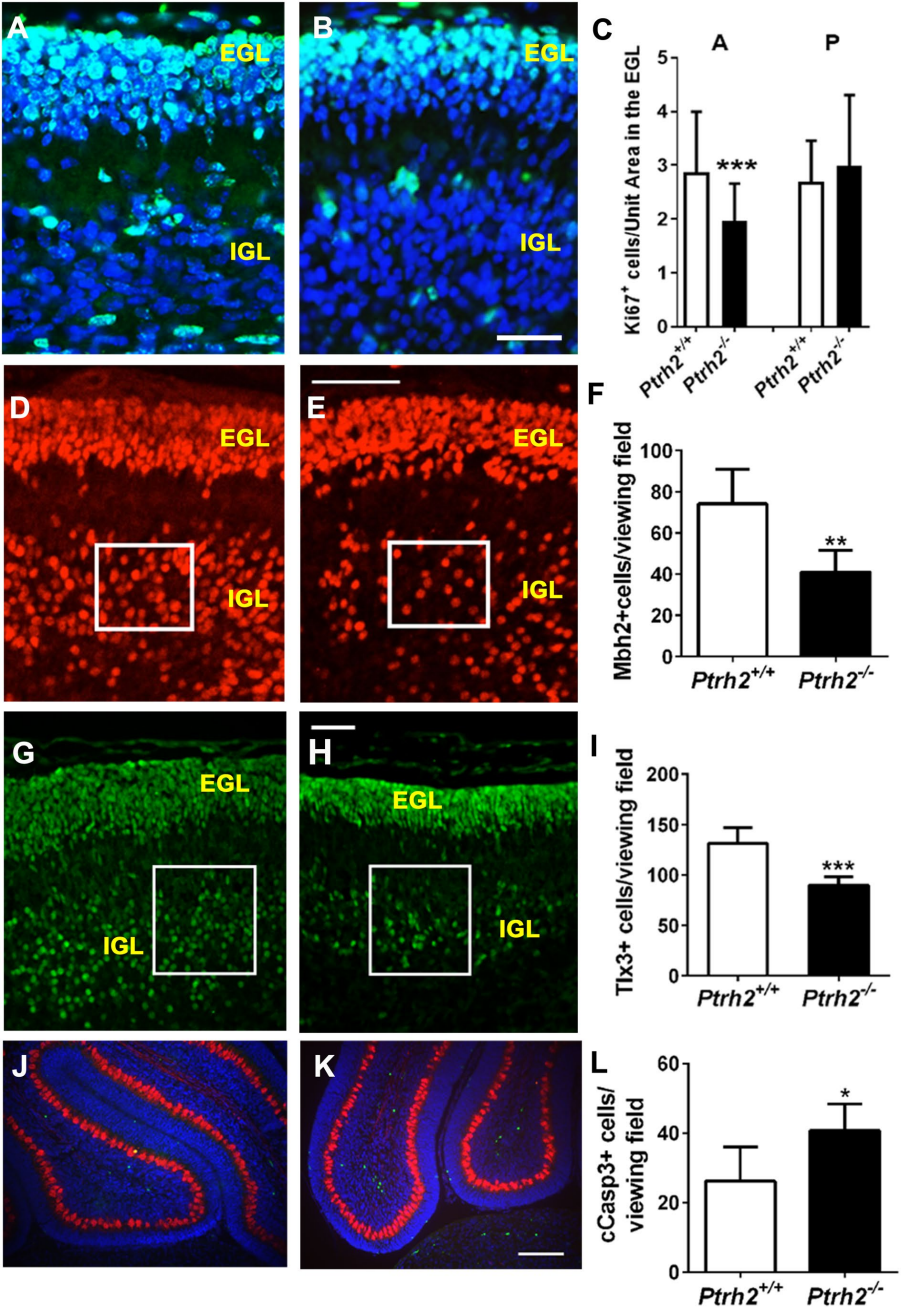
P5 *Ptrh2*^*−/−*^ mice have reduced cell proliferation in the EGL and reduced number of neurons in the IGL. Ki67 IHC (green) in (**A**) *Ptrh2*^+/+^ and (**B**) *Ptrh2*^*−/−*^ cerebellum (scalebar 25 µm). (**C**) Quantitation of Ki67 + cells in the EGL of the anterior (A) and posterior (P) lobes. MBH2 IHC (red) in (**D**) *Ptrh2*^+/+^ and (**E**) *Ptrh2*^*−/−*^ cerebellum (scalebar 50 µm). Box denotes region where cells were counted. (**F**) Quantitation of MBH2 + cells. (positive cells per 2 × 2 cm viewing field). TLX3 IHC (green) in (**G**) *Ptrh2*^+/+^ and (**H**) *Ptrh2*^*−/−*^ cerebellum (scalebar 50 µm). Box denotes region where cells were counted. (**I**) Quantitation of TLX3 + cells in the posterior cerebellum (positive cells per 400 × 600Px viewing field) Calbindin (red) Cleaved caspase3 (green) IHC (**J**) *Ptrh2*^+/+^ and (**K**) *Ptrh2*.^*−/−*^ cerebellum (scalebar 100 µm). (**L**) Quantitation of Caspase 3 + cells in the IGL. Blue = DAPI. Quantitation based on *n* = 6 animals/group. *p < 0.05 **p < 0.01, ***p < 0.0001

Both *Ptrh2*^+*/*+^ and *Ptrh2*^*−/−*^ mice had an external (EGL) and an internal granular layer (IGL) as expected at P5. Analysis of proliferating cells by Ki67 immunohistochemistry (IHC) was done across all cerebellar lobes and revealed a significantly reduced number of proliferating GNPs in the EGL in *Ptrh2*^*−/−*^ mice in lobes anterior to the primary fissure (A in Fig. 2C) but not in the lobes posterior to the primary fissure (P in Fig. 2C) (Fig. 2A–C). *Mbh2* is a Bar-class homeobox gene that is downstream of *Math1* and expressed in all GNPs and GCs [24]. Quantitation showed that the number of MBH2 + GNs in the IGL was reduced in the *Ptrh2*^*−/−*^ mice (Fig. 2D–F). The homeobox gene *Tlx3* is a specific marker for the GNPs and GNs of the posterior lobes of the cerebellum [25]and using this second marker also showed reduced numbers of TLX3 + cells in the IGL of the posterior lobe (Fig. 2G–I). We next analyzed whether there was increased cell death in the cerebella of *Ptrh2*^*−/−*^ mice by activated caspase 3 IHC. We detected an increased number of apoptotic cells in the IGL in the *Ptrh2*^*−/−*^ mice compared to *Ptrh2*^+*/*+^ and mice (Fig. 2J–L).

### PCs Express Mature Differentiation Markers but Are Morphologically Immature in *Ptrh2*.^*−/−*^ Mice at P5

**Fig. 3:**
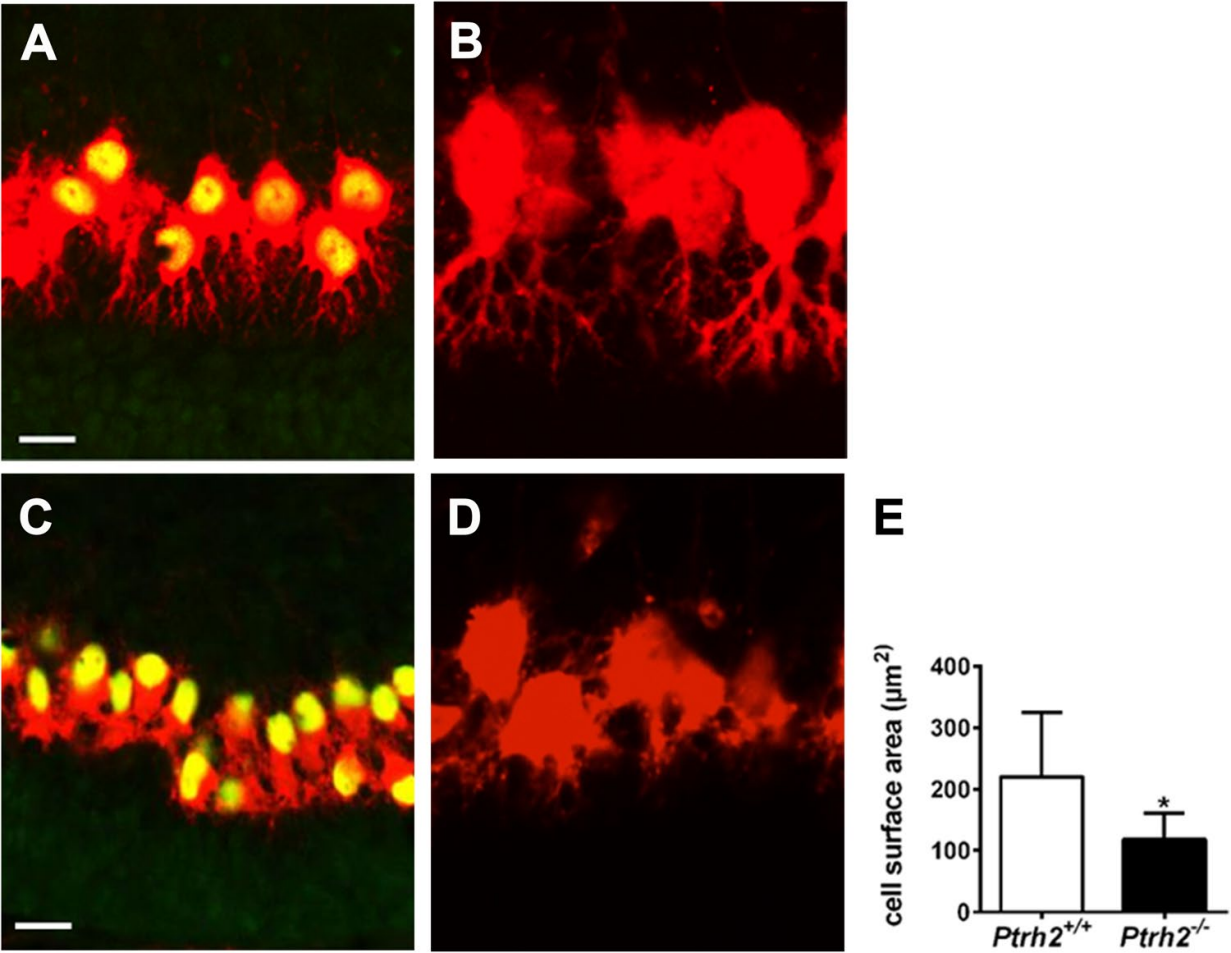
P5 *Ptrh2*^*−/−*^ mice have abnormal PC morphology. IHC for Calbindin (red) and FoxP4 (green) in (**A**) *Ptrh2*^+/+^ and (**C**) *Ptrh2*^*−/−*^ cerebella. Detail of calbindin stained PCs at higher magnification (**B**) *Ptrh2*^+/+^ (**D**) *Ptrh2*.^*−/−*^ cerebella. (a and c: scalebar 50 µm, b and d: detail of PC morphology). (**E**) Quantitation of PC surface area (*n* = 6 animals/group). *p < 0.05

Since we previously showed that PTRH2 was highly expressed in PCs, we next analyzed PC differentiation. PCs were identified by expression of calbindin and FOXP4, a transcription factor that is specifically expressed in migrating and mature PCs [26]. At P5 in *Ptrh2*^+*/*+^ mice, PCs had formed a monolayer and had developed an elaborate network of dendrites (Fig. 3A, B). In contrast, although they expressed both calbindin and FOXP4, PCs in *Ptrh2*^*−/−*^ mice were still multilayered, had a small soma, and morphologically had a simplified shorter dendritic structure (Fig. 3C–E).

### *Ptrh2* Is Required at Early Stages of PC Differentiation

**Fig. 4:**
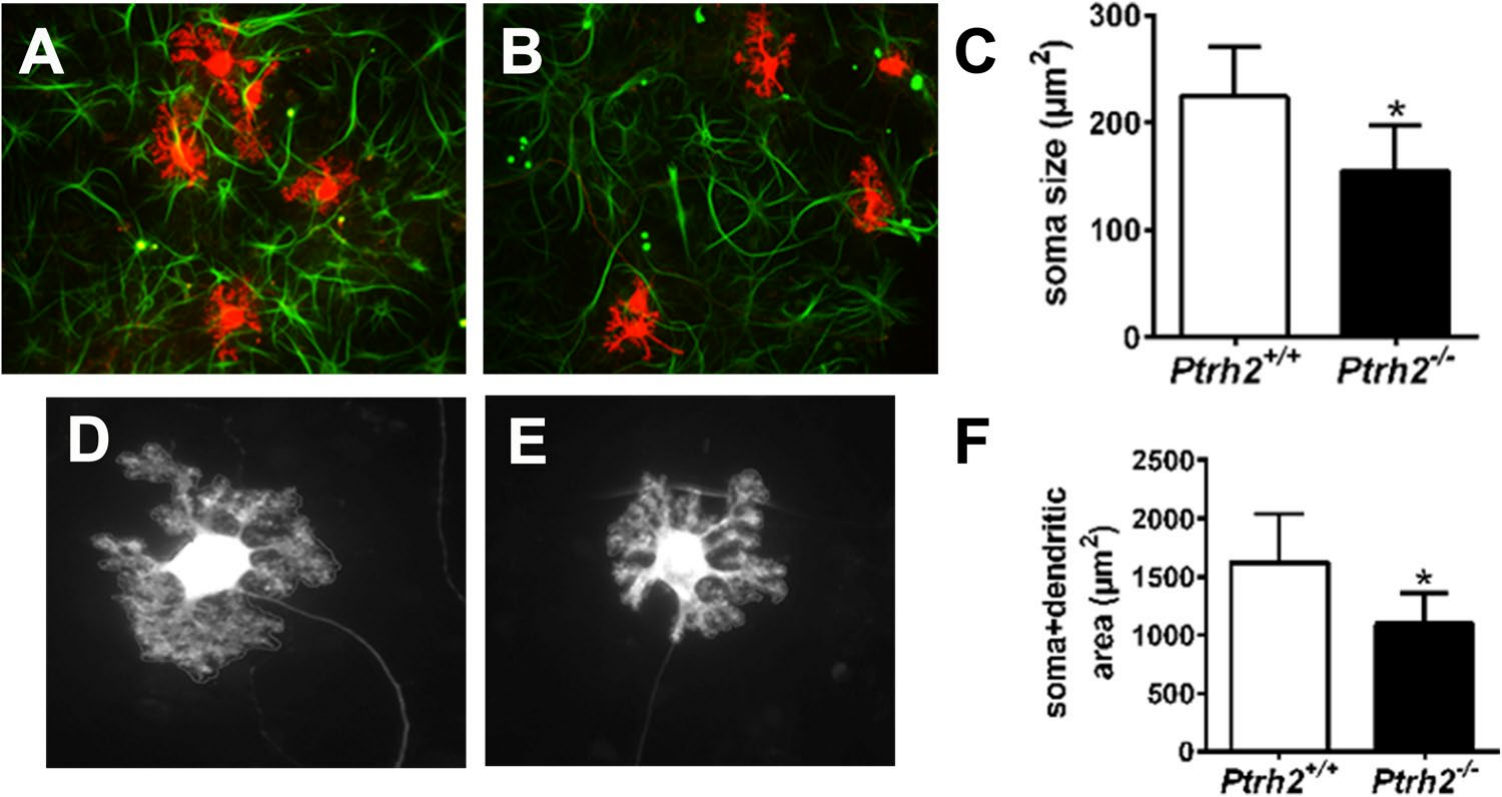
PC culture from *Ptrh2*^*−/−*^ mice are abnormal. IHC for Calbindin (red) and GFAP (green) in DIV12 mixed cerebellar cultures from (**A**) *Ptrh2*^+/+^ (**B**) *Ptrh2*^*−/−*^ mice. (**C**) Quantitation of PC soma size (*n* = 6, number of cells = approx. 60 per genotype). Morphology of PC from DIV12 cultures from (**D**) *Ptrh2*^+/+^ (**E**) *Ptrh2*.^*−/−*^ mice. (**F**) Quantitation of somatic + dendritic area of PCs, *n* = 6 number of cells = approx. 60 per genotype, *p < 0.05

Since *Pcp2-Cre* is not widely active at P1, in order to investigate the role of *Ptrh2* during early stages of PC differentiation, we went to an in vitro system to avoid the effect of malnutrition and dystrophy observed in *Ptrh2*^*−/−*^ mice as a confounding factor. PC structure in vitro was analyzed by generating primary mixed cerebellar neuron cultures from cerebellar lysates of *Ptrh2*^+*/*+^ and *Ptrh2*^*−/−*^ mice at P1 (Fig. 4A, B). We measured the area of soma and dendritic tree as an estimate for PC growth and show reduced size of PC soma (Fig. 4C). Further, quantitation of dendrite growth at DIV12 showed reduced dendritic structure in calbindin positive cells from *Ptrh2*^*−/−*^ mixed cerebellar cultures (Fig. 4D–F) confirming that the loss *Ptrh2* has a disruptive effect on PC development.

### SHH Signaling Is Reduced in *Ptrh2*^*−/−*^Mice

**Fig. 5:**
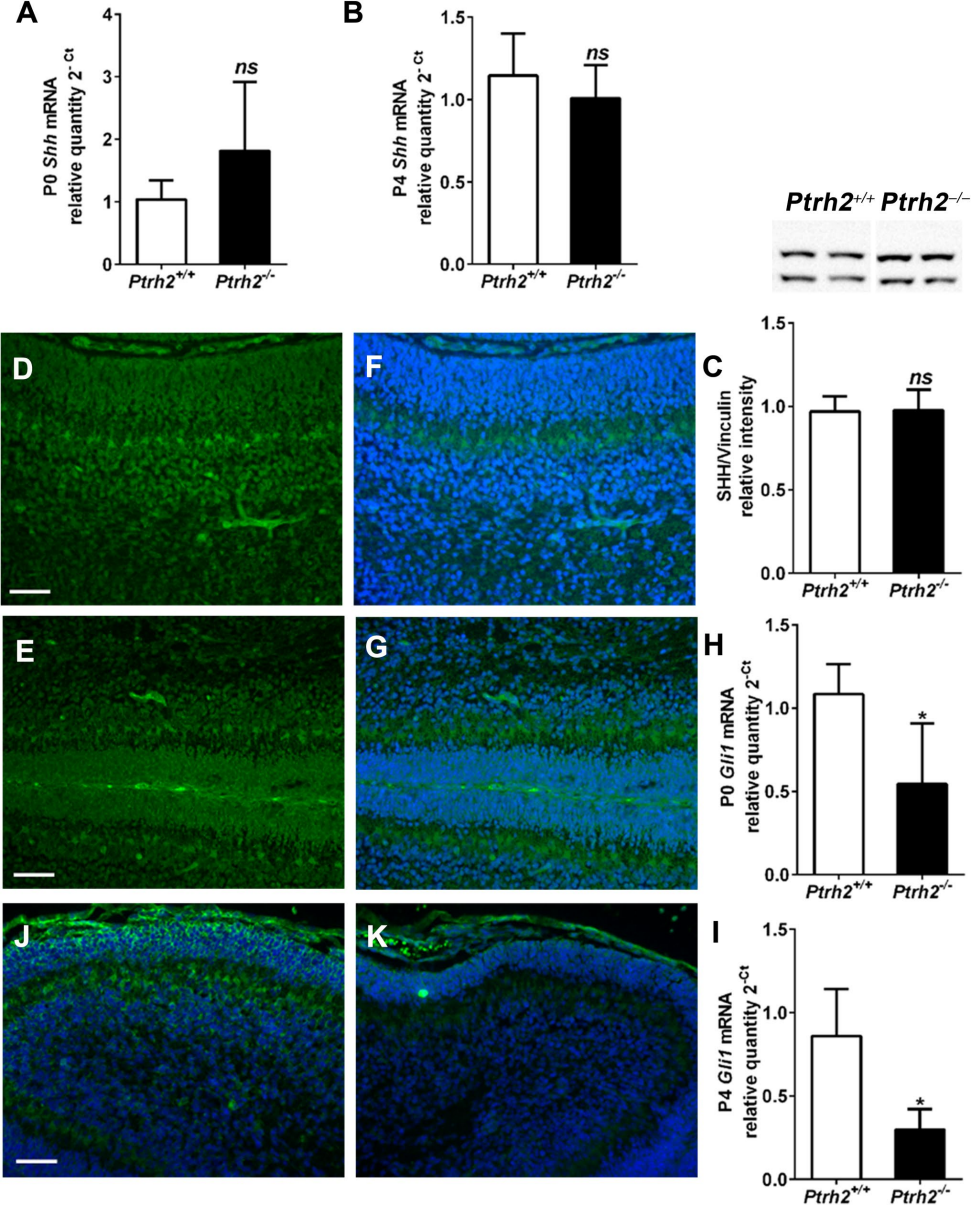
SHH signaling is reduced in the cerebellum of *Ptrh2*^*−/−*^ mice. Quantitative PCR for *Shh* at (**A**) P0 and (**B**) P4 (P0 (*n* = 3/group) and P4 (*n* = 3/group)). (**C**) SHH western blot from P5 cerebellum with representative blot showed above graph (*n* = 3/group). SHH (green) IHC in P5 cerebella (**D**) *Ptrh2*^+/+^ and (**E**) *Ptrh2*^*−/−*^ mice. SHH images merged with DAPI (blue) (**F**) *Ptrh2*^+/+^ and (**G**) *Ptrh2*^*−/−*^ (*n* = 6). Quantitative PCR for *Gli1* at (**H**) P0 and (**I**) P4 (P0 (*n* = 3) and P4 (*n* = 3)). GLI1 (green) IHC merged with DAPI (blue) in P5 cerebella (**J**) *Ptrh2*^+/+^ and (**K**) *Ptrh2*^*−/−*^ mice (*n* = 5). Scalebar 100 µm

SHH signaling in GNPs is required for the proliferation of GNPs, and PCs are the source of SHH [11]. Since we saw a decrease in GNP proliferation and abnormal PC differentiation, we next analyzed SHH signaling. *Shh* mRNA expression in cerebellar lysates of *Ptrh2*^*−/−*^ mice at P0 and P4 was unaltered (Fig. 5A, B). In line with this finding, protein analysis of cerebellar lysates at P5 as well as IHC for SHH on P5 cerebellar sections showed that SHH was unaltered between *Ptrh2*^+*/*+^ and *Ptrh2*^*−/−*^ mice (Fig. 5C–G). In contrast, both gene expression at P0 (Fig. 5H) and P4 (Fig. 5I) and IHC staining for GLI1 on P5 cerebellar sections (Fig. 5J, K) showed a marked reduction of GLI1 in the EGL.

### Adult *Ptrh2*^*ΔPC*^ Mice Show Cerebellar Atrophy and Loss of PC

**Fig. 6:**
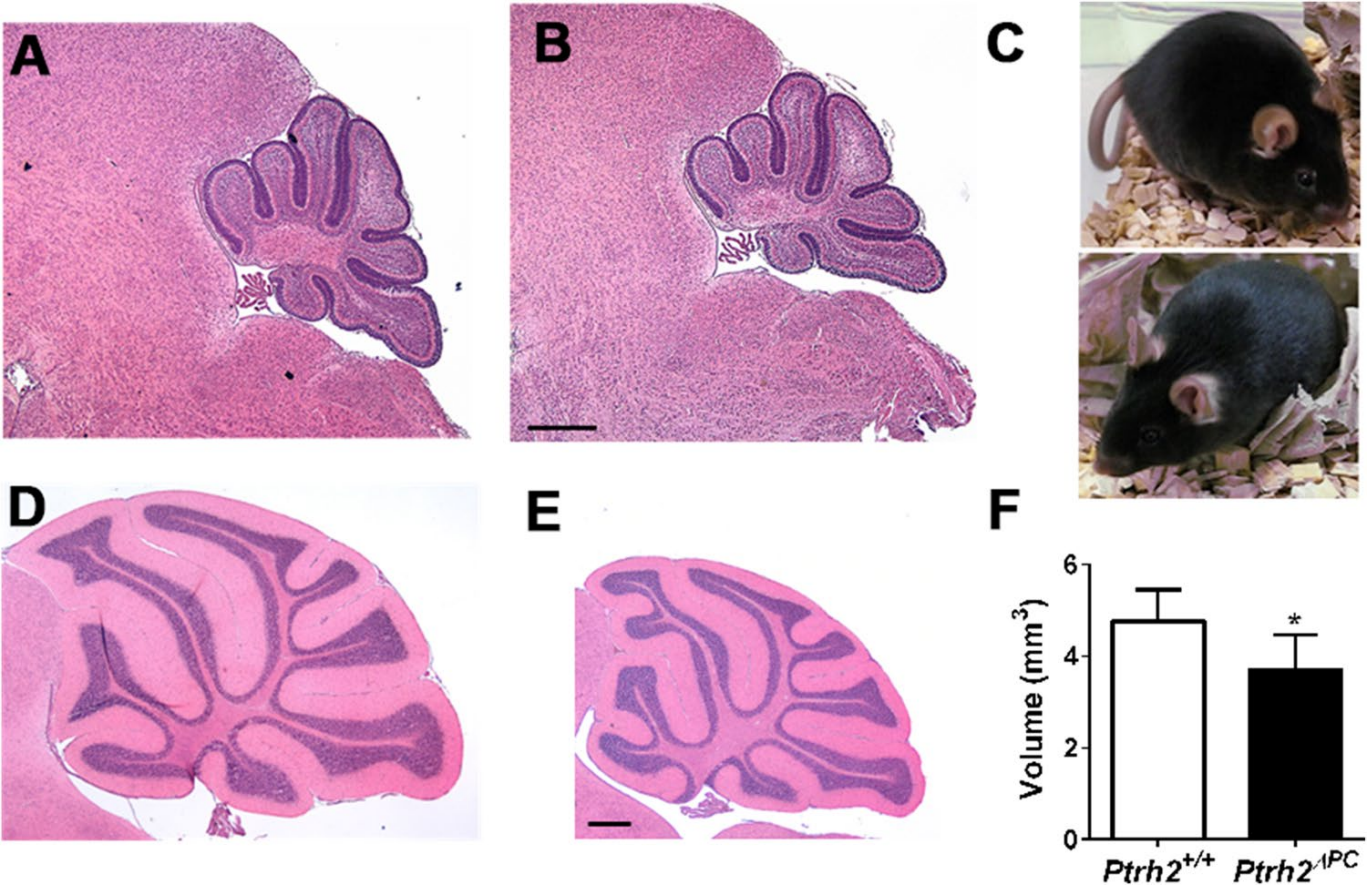
*Ptrh2*^ΔPC^ mice show adult cerebellar atrophy. H&E staining of midsagittal section at P5 showing cerebellum of (**A**) *Ptrh2*^+/+^ and (**B**) *Ptrh2*^ΔPC^ mice. (**C**) *Ptrh2*^+/+^ (top) and *Ptrh2*^ΔPC^ (bottom) mice at 8 weeks. H&E staining of midsagittal section at 8 weeks (**D**) *Ptrh2*^+/+^ and (**E**) *Ptrh2*.^ΔPC^ (H&E, scalebar 400 µm) (**F**) quantitation of cerebellar volume (*n* = 5) *p < 0.05

In contrast to *Ptrh2*^*−/−*^ mice, *Ptrh2*^*ΔPC*^ mice were indistinguishable from *Ptrh2*^+*/*+^ littermates and had normal cerebellar volume and gross morphology at P5 (Fig. 6A, B). At adult age of 8 weeks, *Ptrh2*^*ΔPC*^ mice appeared healthy and were phenotypically unremarkable (Fig. 6C). However, histological analysis of their cerebella demonstrated significant cerebellar atrophy with reduced cerebellar volume (Fig. 6D–F).

**Fig. 7:**
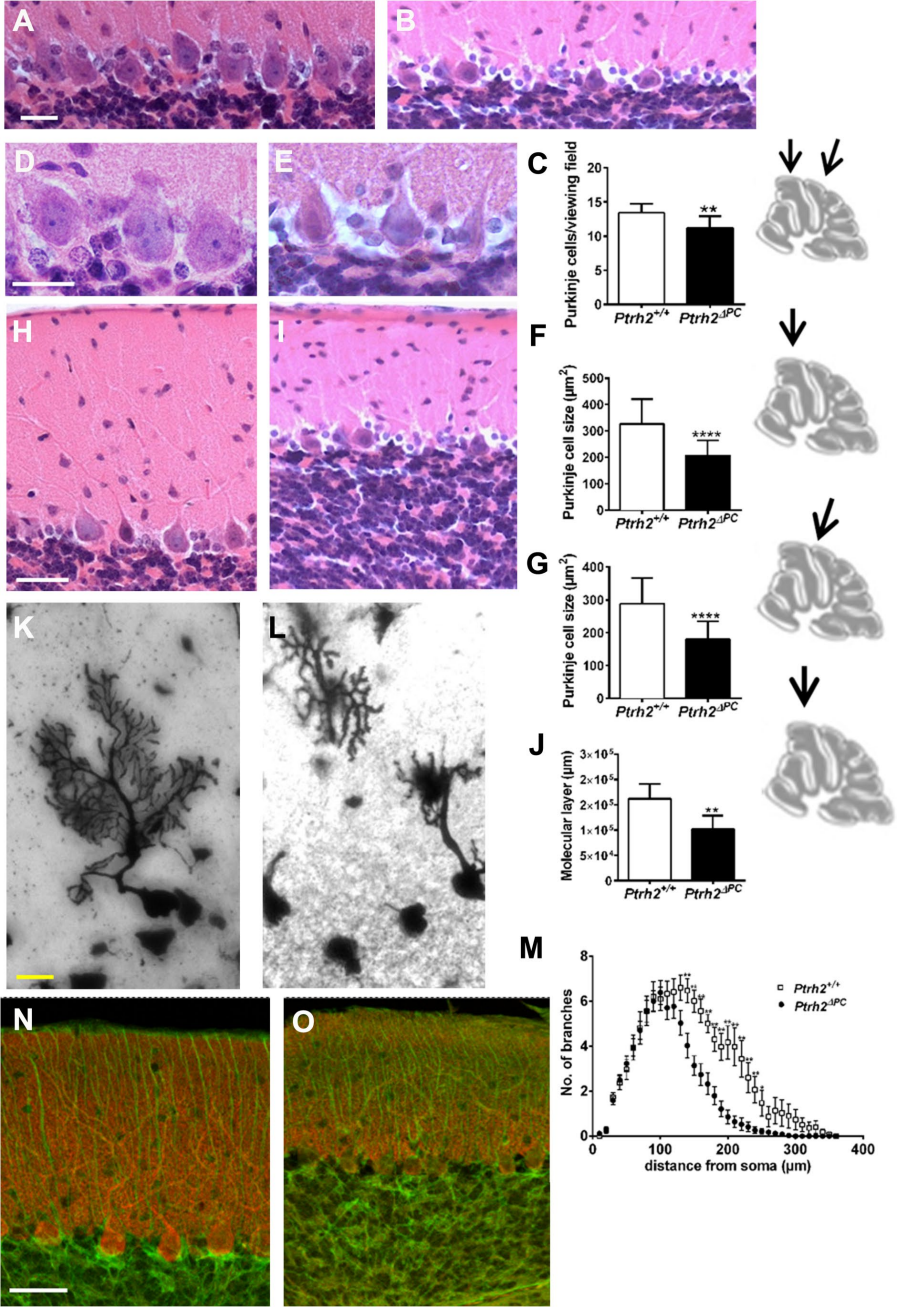
Purkinje cell degeneration in the adult *Ptrh2*^ΔPC^ mice. H&E staining showing PC layer in (**A**) *Ptrh2* ^+/+^ and (**B**) *Ptrh2*^ΔPC^ mice. (**C**) Quantitation of cell density in two different areas (scalebar 20 µm). H&E staining for PC soma quantitation (**D**) *Ptrh2* ^+/+^ and (**E**) *Ptrh2*^ΔPC^ mice (**F** and **G**) quantitation of PC soma size in two different lobes indicated by black arrow (scalebar 20 µm). H&E staining of molecular layer in (**H**) *Ptrh2* ^+/+^ and (**I**) *Ptrh2*^ΔPC^ mice (scalebar 20 µm). (**J**) Quantitation of height of the molecular layer. (*n* = 6/group). Golgi staining of a PC in (**K**) *Ptrh2* ^+/+^ and (**L**) *Ptrh2*^ΔPC^ mice and (**M**) Sholl analysis of dendrites (*n* = 30 cells/6 animals, mean ± SEM; *p < 0.05, **p < 0.01). Calbindin (red) and GFAP (green) IHC in 8-week-old (**N**) *Ptrh2* ^+/+^ and (**O**) *Ptrh2*.^ΔPC^ mice. (Scalebar 50 µm). *p < 0.05, **p < 0.01, ***p < 0.0001

At the cellular level, PC density (Fig. 7A–C) and soma size (Fig. 7D–G) were reduced in the adult *Ptrh2*^*ΔPC*^ mice cerebellum. The thickness of the molecular layer, consisting mainly of the dendrites of the PCs, was also strongly reduced (Fig. 7H–J). We performed Golgi staining to analyze the structure and morphology of the dendritic tree in more detail and showed reduced complexity of the dendritic tree in the *Ptrh2*^*ΔPC*^ mice (Fig. 7K, L). The number of branches of the dendritic tree close to the soma were similar to the *Ptrh2*^+*/*+^ mice, but branching was significantly affected at further distance from the soma (Fig. 7M). In 18-month-old animals, IHC staining for calbindin to visualize PC soma and dendrites and GFAP to visualize Bergmann glia showed marked loss of PCs especially in the anterior lobe, and the GFAP staining in the molecular layer of *Ptrh2*^*ΔPC*^ mice suggests that there might be an increase (Fig. 7N, O).

### *Ptrh2*^*ΔPC*^ Mice Develop Progressive Cerebellar Ataxia 

The *Ptrh2*^*ΔPC*^* mice* survived throughout our observation period of 18 months and were phenotypically indistinguishable from *Ptrh2*^+*/*+^ littermates with no difference in weight at 18 months.

**Fig. 8:**
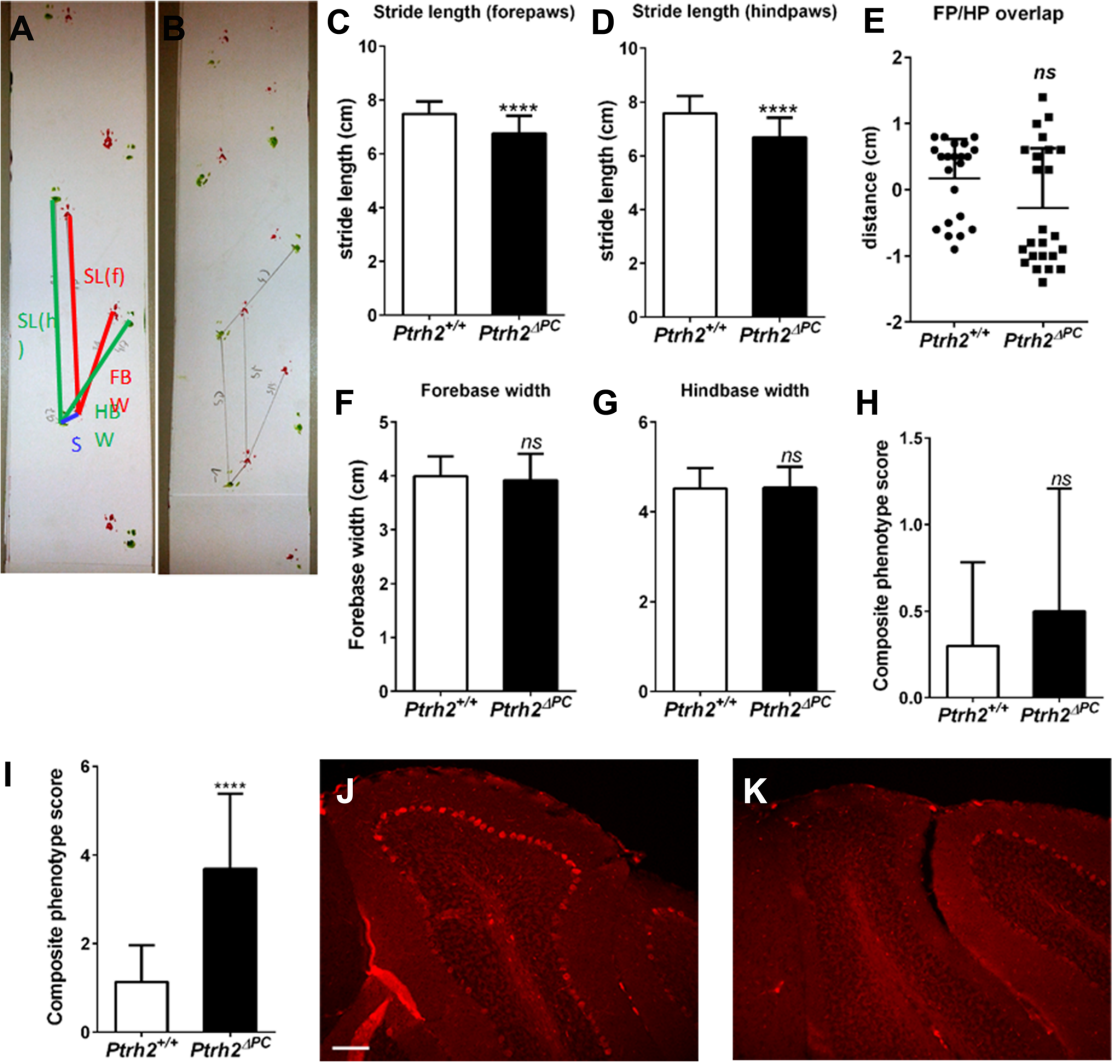
Adult *Ptrh2*^ΔPC^ mice develop motor deficits. Footprint analysis in 6-month-old mice. Footprint pattern in (**A**) *Ptrh2* ^+/+^ and (**B**) *Ptrh2*^ΔPC^ mice. Stride length of (**C**) forepaws and (**D**) hindpaws. (**E**) Forepaw (FP)/hindpaw (HP) overlap. (**F**) Forebase width, (**G**) hindbase width (*n* = 22). Composite phenotype score of (**H**) 6-month-old mice (*n* = 9) and (**I**) 18-month-old mice (*n* = 15). Calbindin IHC of 18-month-old mice (**J**) *Ptrh2* ^+/+^ and (**K**) *Ptrh2*.^ΔPC^ (scalebar 100 µm). (*ns*, not significant *p < 0.05, **p < 0.01, ***p < 0.0001)

We evaluated their motor function at 6 months and at 18 months using different measures. At the age of 6 months, *Ptrh2*^*ΔPC*^ and *Ptrh2*^+*/*+^ littermates were phenotypically indistinguishable, and no apparent movement abnormalities were noted on routine observation. In a footprint analysis, however, we identified a significantly reduced stride length in *Ptrh2*^*ΔPC*^ (Fig. 8A–D). Further features like sway and stance length were unaltered (Fig. 8E–G). We additionally performed a composite phenotype scoring system for cerebellar ataxia [23] including a ledge test and measurement of hind limb clasping, gait, and kyphosis at the age of 6 months and 18 months. At the age of 6 months, there was no significant difference in the composite phenotype scoring test (Fig. 8H). However, at 18 months the *Ptrh2*^*ΔPC*^ mice presented with visible tremor, head circling and instable gait. The composite phenotype scoring system revealed significantly higher score indicating ataxia in the *Ptrh2*^*ΔPC*^ mouse (Fig. 8I). In summary, we here show that the progressive cerebellar atrophy in *Ptrh2*^*ΔPC*^ mice impairs motor function and causes ataxia. Further calbindin staining in 18-month-old animals shows that there was an almost complete loss of PCs especially in the anterior lobes (Fig. 8J, K).

### Cerebellum of *Ptrh2*^*ΔPC*^ Mice Shows Reduced pS6 Expression 

**Fig. 9:**
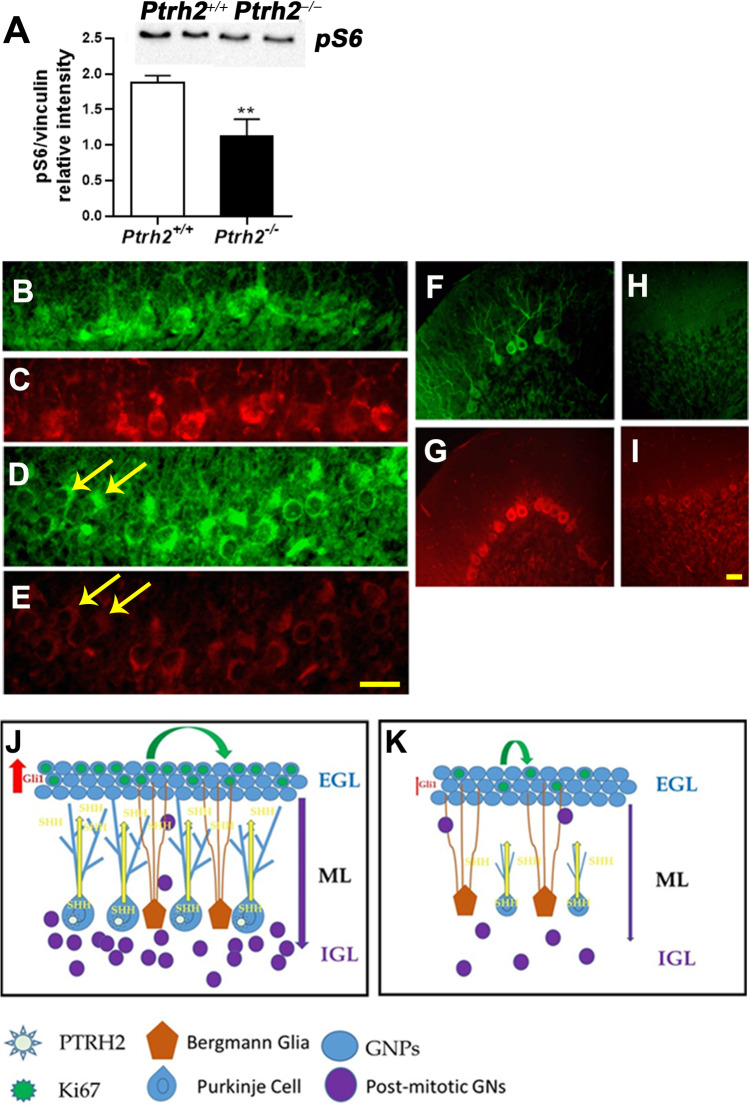
Downregulation of pS6 in *Ptrh2*^*−/−*^ and *Ptrh2*^ΔPC^ mice. (**A**) Western blot for pS6 from P5 cerebella with representative blot shown above. IHC for Calbindin (green) and pS6 (red) in P5 cerebellum (**B**–**C**) *Ptrh2* ^+/+^ (**D**–**E**) *Ptrh2*^ΔPC^. Yellow arrows in (**D**) and (**E**) point to cells that have downregulated pS6. IHC for calbindin (green) and pS6 (red) in adult cerebellum (**F** and **G**) *Ptrh2* ^+/+^ (**H**–**I**) *Ptrh2*^ΔPC^ mice. Scalebar 50 µm. **p < 0.01. Model of cerebellar development in *Ptrh2*^*−/−*^ and *Ptrh2*^ΔPC^ mice. (**J**) *Ptrh2* expressing PCs are morphologically mature, and the secreted Shh reaches the outer EGL causing Gli mediated signal transduction and proliferation in the GNPs. Post-mitotic GNs migrate along glial fibers to form the IGL layer. (**K**) In the absence of *Ptrh2* expression in PCs, they are morphologically stunted, and although there is no difference in the level (depicted by the yellow arrow width), the Shh morphogenetic gradient (depicted by the length of the yellow arrow) is diminished causing lower Gli mediated signal transduction and proliferation. This leads to lower numbers of GNs in the IGL

The mTOR pathway is critical for PC survival, and we had previously reported that pS6, a readout of the mTOR pathway activation was downregulated in the *Ptrh2*^*−/−*^ mouse line *Ptrh2LoxPxMeoxCre* [1]. We show here by western blot that pS6 protein is downregulated in the cerebellum of *Ptrh2*^*−/−*^ mice at P5 (Fig. 9A). We next performed IHC for pS6 and demonstrate that at P5, when the *Pcp2-Cre* recombination is still mosaic, many PCs in the *Ptrh2*^*ΔPC*^ mice have lower expression levels of pS6 (9D and 9E, arrows) compared to *Ptrh2*^+*/*+^ mice (Fig. 9C). As described above, the PCs degenerate in the adult *Ptrh2*^*ΔPC*^ cerebellum. Nevertheless, IHC showed that compared to the *Ptrh2*^+*/*+^ cerebella, where prominent expression of pS6 is seen (Fig. 9G), the few remaining PCs firstly showed a striking loss of dendritic arbors and markedly lower levels of pS6 expression (Fig. 9H, I). One potential model for cerebellar development in the presence (Fig. 9J) and absence (Fig. 9K) of PTRH2 expression in PCs is shown. The main findings are taken into account in this model namely a decrease in cell proliferation in the EGL, a decrease in the thickness and density of the IGL, lower levels of Gli1, and the stunted morphology of PCs.

## Discussion

PTRH2 is expressed in postmitotic neurons including PCs in the developing cerebellum, and in the adult cerebellum is specific to PC soma and dendrites [1]. Whether *Ptrh2* function is required in mature PCs is not known since *Ptrh2*^*−/−*^ mice are runted and die during the period when PCs are undergoing dendritic growth and synaptic remodeling. In this study, we generated a conditional knockout where expression of *Ptrh2* is deleted in all PCs after the first postnatal week and show that survival of PCs is critically dependent on cell autonomous *Ptrh2* function in PCs. This study strongly suggests that the underlying cause of ataxia and cerebellar atrophy seen in IMNEPD patients is due to the cell autonomous role that *PTRH2* plays in PC maturation and survival.

Although *Ptrh2* has been shown to play a role in cellular differentiation in other contexts [27], we show that *Ptrh2* is not required for the early differentiation and migration of postmitotic neurons from the VZ which occurs before birth [28]. At P0, cerebella of *Ptrh2*^+*/*+^ and the *Ptrh2*^*−/−*^ mice are comparable, both also showing the beginning of folia invagination and cardinal lobe formation. PCs begin to disperse and form a monolayer around P5 that is a peak period for GNP proliferation [11]. At this stage, SHH is required to maintain the GNP proliferative pool in order to generate sufficient numbers to achieve normal lobule growth [29]. Therefore, the foliation defects seen are most probably a secondary consequence of impaired GNP proliferation. High levels of SHH signaling lead to an increase in activated GLI2 which results in the transcription and translation of *Gli1*, and thus GLI1 expression is a readout of SHH signaling levels [30]. In line with this, our data show that at both P0 and P5, although SHH level is normal GLI1 level is lower in *Ptrh2*^*−/−*^ mice. One potential explanation for normal SHH levels in PCs but lower GLI1 levels in the EGL could be that in the developing cerebellum, SHH tethers to PC body, dendrites, and axons with short range SHH signaling playing an important role. Therefore, not only the absolute levels of SHH but also the localization of PC-tethered SHH and the SHH gradient across the cerebellum will have an effect on GNP proliferation [11, 31, 32]. Thus, stunted cell body and dendrites would limit the availability of SHH to GNPs in the EGL even if the level of SHH produced by PCs is not affected, and this would be reflected in GLI1 transcriptional levels.

Taken together, this suggests that lobe formation starts equally at P0 in *Ptrh2*^*−/−*^ mice, but the folia do not progress normally when further development of folia is driven primarily by SHH-dependent GNP proliferation. The lower levels of Ki67 and Gli1 in *Ptrh2*^*−/−*^ mice substantiate this interpretation. The consequent reduction in cerebellar area also possibly exacerbates the inability of PC to form a monolayer like seen in the *Gli*2 KO mice [29]. Collectively, our results show that in the absence of *Ptrh2*, PCs do not form a monolayer, have a smaller soma size and dendrites, and are not able to effectively signal to the overlying EGL because of the lack of morphological differentiation.

Interestingly, *Ptrh2*^*ΔPC*^ mice in contrast to the *Ptrh2*^*−/−*^ mice do not show marked defects at P5 when compared to *Ptrh2*^+*/*+^ mice. This can be explained by the fact that *Pcp2-Cre* recombination is initially mosaic and widespread recombination in PCs and therefore deletion of *Ptrh2* in PCs occurs only around P6 [11, 33]. In addition, this also suggests that the defects seen at P5 in *Ptrh2*^*−/−*^ mice could be a consequence of the deletion of expression of *Ptrh2* in other postmitotic neurons in the IGL and deep cerebellar nuclei.

Our most salient result is to show the progressive atrophy of PCs in the *Ptrh2*^*ΔPC*^ mice with age. In the adult, we see severe cerebellar atrophy and almost a complete loss of PCs especially in the anterior lobes. Excitatory input via GN parallel fibers (PFs) are important for the survival of PCs, and the caudal lobes are less dependent on SHH signaling from the PC layer; thus, the SHH-independent survival of GNs in the posterior lobe could contribute to the better survival of PCs in the posterior lobe [11, 34]. This gradual degeneration of the PCs cannot simply be explained as the secondary effect of abnormal PF connections since in the *Gli2* KO PCs are still present at 6 months [29]. Even in the *Shh* KO, there is only a loss of 65% of PC in adulthood due to loss of trophic support from the GNs [11].

Previous studies have shown that the mTOR pathway is required for the survival of PCs. Disruption of this signaling pathway results in defects in foliation and severe morphological changes in PCs including a reduction in cell size, dendritic arbor, and progressive loss of PCs [7–9]. This was accompanied by astrogliosis as we also see in the *Ptrh2*^*ΔPC*^ mice in the current study [7, 35]. pS6 is activated by mTORC1, and thus pS6 level is a reliable marker of mTOR activation [7, 9]. Here, we show a decrease in pS6 protein in *Ptrh2*^*ΔPC*^ cerebella, indicative of mTOR activity being downregulated. Thus, our results strongly suggest that *Ptrh2* regulates the mTOR pathway in PCs, and disruption of this signaling pathway leads to the loss of PCs. Although PTRH2 has been shown to modulate the PI3K/AKT pathway [36], our previous study did not show a significant difference in pAKT levels in either the patient fibroblasts nor in *Ptrh2-Meox-Cre* mice [1]. Ptrh2 is a peptidyl-tRNA hydrolase present in the mitochondria and important for efficient translation by hydrolyzing aberrant peptidyl-tRNA [36]. Since mTOR controls both protein synthesis and mitochondrial processes that provide energy for this process[7], Ptrh2 may play a critical role during PC maturation, a process with higher energy requirement [37].

PCs form the sole output of the cerebellar cortex, and PC pathology is highly linked to ataxia phenotype [10]. Disruption of PC maturation and survival due to mTOR deactivation has functional consequences including an imbalance between excitatory and inhibitory inputs [7, 38]. The development of the PFs is important for the growth and arrangement of the mature dendritic development in PCs [13]. Here, we show that there are a smaller number of mature GNs in the IGL, and, thus, impairment of PF synapses could lead to the motor impairment seen here [39]. In addition, although the early phase of climbing fiber (CF) refinement is not dependent on proper generation of GNs and PF-PC connections, the late phase of CF refinement is dependent on excitatory PF synapses which could be defective due to the decrease in mature GNs. Thus, in the patients where we have previously shown a decrease in pS6 and thus mTOR signaling [1], defects in establishment of proper circuitry could occur and result in ataxia even in the absence of obvious cerebellar atrophy [28, 40].

The atrophy of PCs has a further important consequence. It has been shown that the cerebellum has compensatory mechanisms to recover from the loss of GNPs, but this recovery crucially depends on Shh-Gli2 pathway [30]. In the absence of the SHH signaling pathway, there will be a progressive degeneration of the cerebellum as is seen in our *Ptrh2*^*ΔPC*^ mice.

## Conclusion

Given the important role of the cerebellum in both motor and cognitive tasks, our study shows that a gradual loss of PCs due to downregulation of mTOR in the absence of *Ptrh2* could result in compromised cerebellar function leading to both motor and non-motor symptoms that are seen in IMNEPD patients. It has been reported that mTOR1 agonists can rescue the cell body size of PC [8, 41] and that enhancement of mTOR signaling can improve motor coordination [42]. Thus, our result indicating that *Ptrh2* is required to activate the mTOR pathway in PCs points to a potentially important therapeutic avenue for these patients.

### Supplementary Information

Below is the link to the electronic supplementary material.

Supplementary file1 (PDF 940 KB)
