# Supplementary material for: PTRH2 is Necessary for Purkinje Cell Differentiation and Survival and its Loss Recapitulates Progressive Cerebellar Atrophy and Ataxia Seen in IMNEPD Patients
Source: Cerebellum. 2022 Oct 11;22(6):1137–51. doi: 10.1007/s12311-022-01488-z (PMC10657312; doi:10.1007/s12311-022-01488-z)
Supplement: Supplementary file 1 — Supplementary file1 (PDF 940 KB) [file 12311_2022_1488_MOESM1_ESM.pdf]

Reviewer #2: question 1

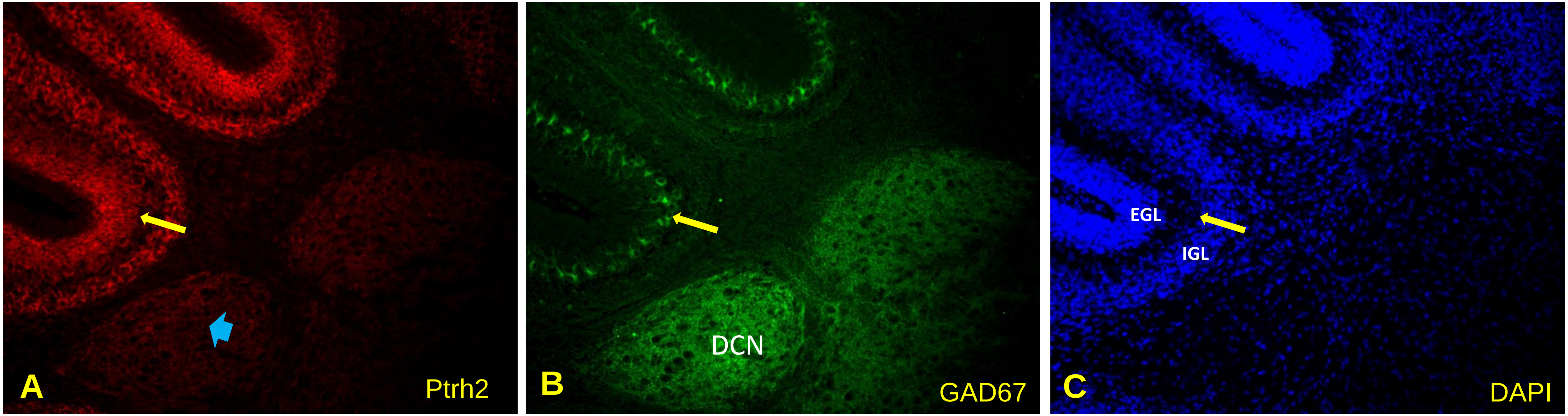

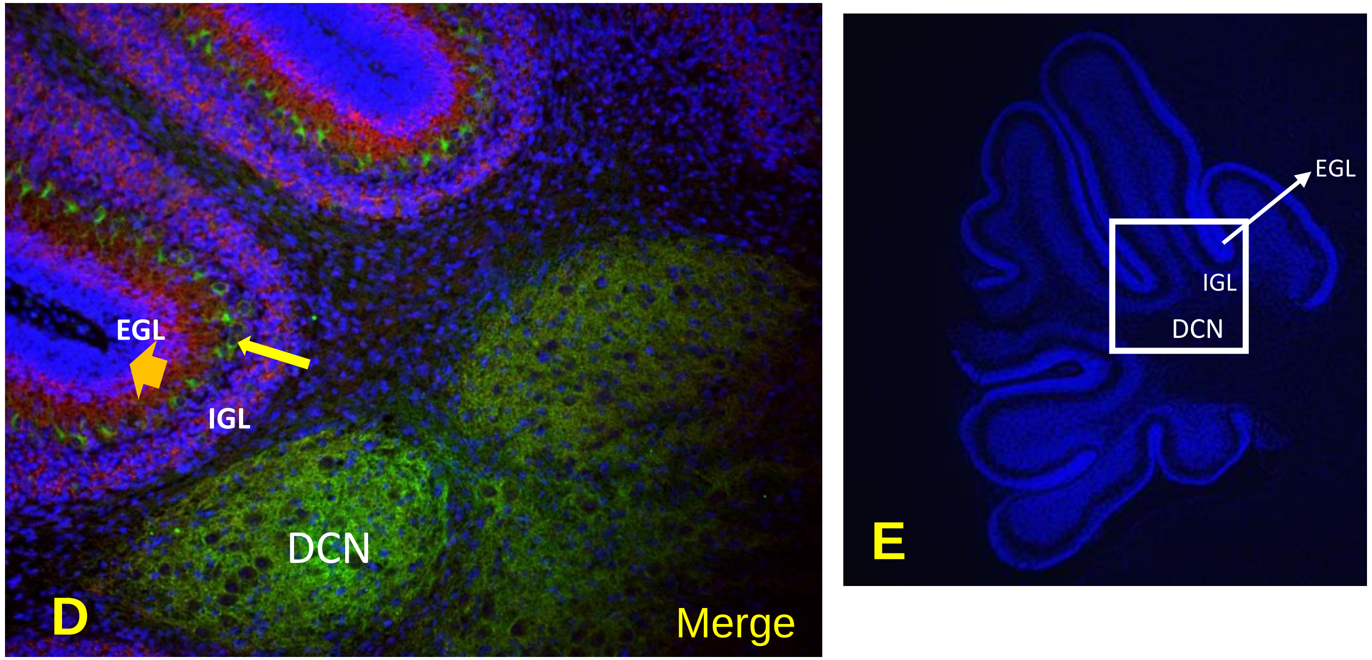

Expression of PTRH2 in P5 cerebellum

It is strongly expressed in the PCs (yellow arrow) and IGL but is not expressed in the EGL (orange short arrow in D). It is expressed at lower levels in the the deep cerebellar nuclei (blue short arrow in A). Deep cerebellar nuclei (DCN) are stained with glutamic acid decarboxylase (GAD67 Millipore MAB5406). E) Low power 4x of the section showing the region from which the image was taken.

Reviewer #2: question 3

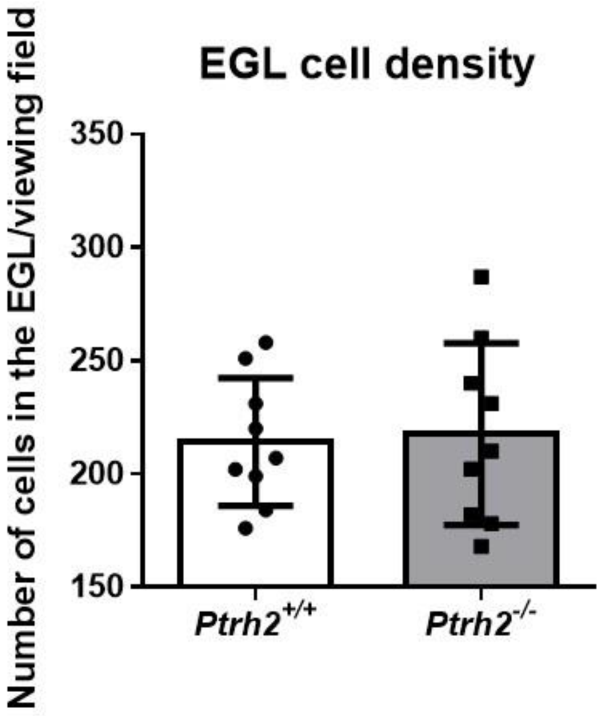

EGL cell density is not changed

Reviewer #2: question 4

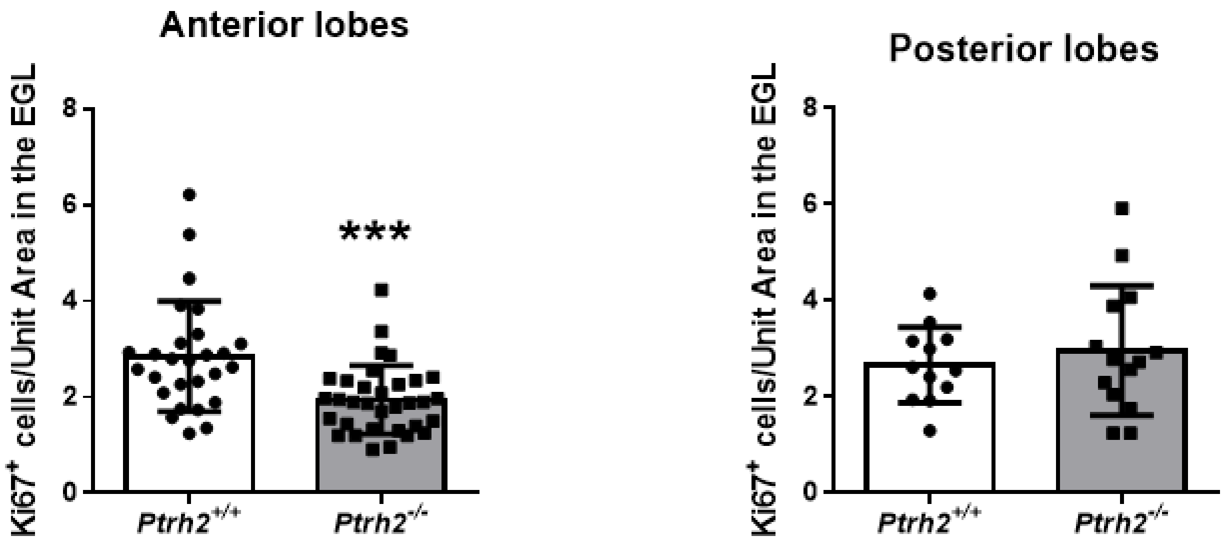

Reviewer #3: question 2

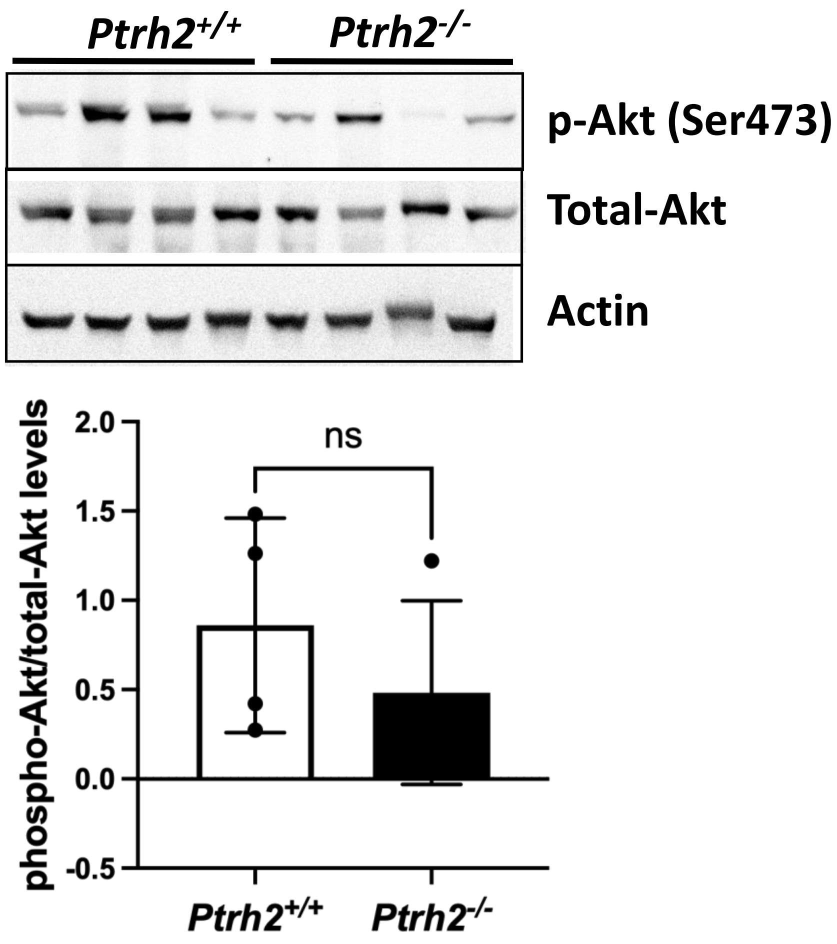

Phospho-Akt (Ser473) Cell signalling#4060 (1:1000)
Total Akt, Cell signalling #9272 (1:1000)
Actin, Millipore, MAB1501 (1:100000)

Reviewer #3: question 2

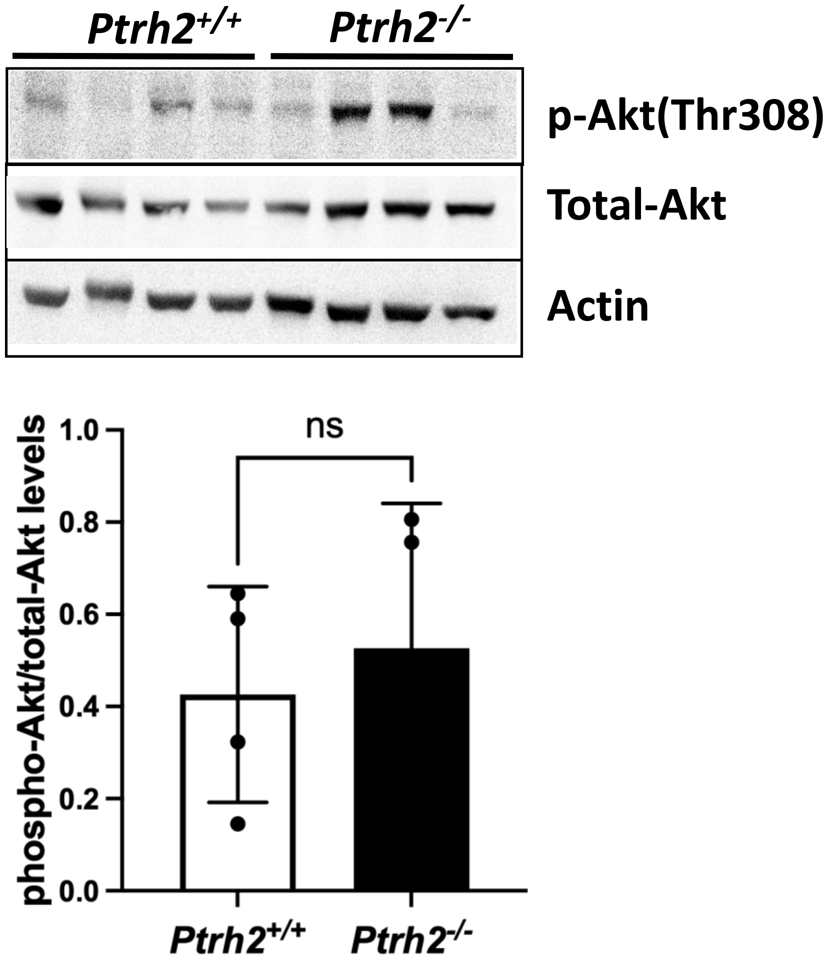

Phospho-Akt (Thr308) Cell Signaling #9275 (1:1000)
Total Akt, Cell signalling #9272 (1:1000)
Actin, Millipore, MAB1501 (1:100000)
